# Resolution of adhesive small bowel obstruction with a protocol based on Gastrografin administration

**DOI:** 10.25122/jml-2018-0082

**Published:** 2019

**Authors:** Abhilash Paily, Jalpa Kotecha, Loveena Sreedharan, Bhaskar Kumar

**Affiliations:** Norfolk and Norwich University Hospitals NHS Foundation Trust, Norfolk, England

**Keywords:** adhesive, obstruction, Gastrografin, bowel

## Abstract

The use of Gastrografin may have a therapeutic effect on resolving adhesive small bowel obstruction.

Adhesive Small Bowel obstruction (ASBO) accounts for the majority of patients with small bowel obstruction. Most patients are managed conservatively; frequent admissions create a considerable burden. We sought to examine the adherence to the Bologna guidelines for the management of ASBO in a high volume tertiary center and whether or not Gastrografin had a therapeutic effect.

A comparison was made between an initial retrospective audit looking at ASBO and a prospective re-audit after applying standards derived from the Bologna guidelines. During re-audit it was found that more patients underwent conservative management and fewer patients had surgery as first line management. In the re-audit, those who had to undergo surgery within/after a period of 72h of conservative management were also fewer. Whether they were managed surgically primarily or after a period of conservative management, the average length of stay was also shorter. In comparison to the preliminary audit, there appeared to be no change in the way that medical history and physical examination was documented during the re-audit. However, there was a marked difference in the use of appropriate blood tests and CT scans.

Changes were made successfully following the initial audit results and have been implemented, thus closing the audit loop. This study shows that the use of Gastrografin has decreased the need for surgical intervention in a group of patients with small bowel obstruction.

## Introduction

Adhesive Small Bowel Obstruction (ASBO) accounts for 65-75% of cases of small bowel obstruction [1,2]. It is a recognized complication of open pelvic and abdominal surgery [3]. Although the majority of these patients are managed successfully by the conservative treatment, they will remain in the hospital for an average of ten days [4]. ASBO is associated with significant morbidity from repeated hospital admissions and diminished quality of life [4–6]. Conservative treatment may fail after several days, and surgical intervention may be required which can result in further adhesions over time [7]. Some preventative measures such as adhesive films have been tried in the past but are generally considered futile. The advent of laparoscopic surgery has reduced adhesion formation but has a limited role in many emergency operations and complex procedures.

As a novel approach, Gastrografin, a water-soluble contrast medium, was used in the management of adhesive small bowel obstruction with promising results [8]. As a result, this approach has been incorporated into the Bologna guidelines for ASBO management [9].

We sought to examine the adherence to the Bologna guidelines for the management of ASBO in a high volume tertiary center. In addition, we examined whether or not Gastrografin had a therapeutic effect on resolving ASBO.

## Materials and Methods

A retrospective analysis of patients admitted with ASBO between July 2013 and July 2014 was conducted. Seventy-two patients were included in the audit, and patient case notes were then obtained. Additional information including laboratory blood results, CT scan reports and operation notes were retrieved from password-protected databases. After presenting our results to our department, we sought to introduce an intervention aimed at increasing adherence to the guidelines. We then prospectively re-audited patients admitted with ASBO using the same standards. A time period of two months was allowed before the re-audit to enable staff to become familiar with the guidelines introduced by our intervention. Sixty consecutive patients with a clinical and radiological diagnosis of ASBO over 6 months from August 2015 to February 2016 were then identified for the re-audit.

The five standards from the guidelines were identified. These were whether there had been adequate documentation of medical history, adequate documentation of physical examination, appropriate blood tests and imaging tests, appropriate conservative management and appropriate surgical management (Table 1). If there was no written documentation, it was assumed that the examination, test or procedure was not done. The guidelines define the failure of conservative management as a surgical intervention after 72 hours of conservative management.

**Table 1:** Standards

Standard	Detail	Target
Adequate documentation of patient history	Bowel opening, nausea vomiting, pain, previous surgery (including date)	100%
Adequate documentation of physical examination	Abdominal examination (including hernial orifices and digital rectal/stomal exam)	100%
Investigations: blood tests and scans	WCC, lactate, U+e’s, AXR, N.B. OT not an absolute recommendation	100%
Appropriate conservative management	All pts should have IV fluids, insertion of NG tube with documentation of output and adminstration of watersoluble contrast medium on admission +/- at 48 hours	100% (exception – GASTROGRAFIN ALLERGY CONTRA-INDICATIONS TO NG INSERTION)
Appropriate surgical management	Pts who are intially conservatively managed should be surgically manged if there is no resolution goes 48 to 72 hours. Specific indication NG output ~500ml/24 hrs on day 3	100%
	2er post gastrografin AXR showing no contrast in colon Conservative mangement can be prolonged in certain cases with close monitoring.	
	Indications of immediate surgical management strangulation, peritonitis, carcinomatosis, irreductible hernia surgery within 6 weeks before acute admission	

## Results

### Retrospective audit

Exploring aspects of diagnosis (Standards 1, 2 and 3, Table 1), 18% of patients (n=13) had documentation of the previous abdominal/pelvic surgery in the history taken. There was no record of bowel movements in 1 patient (1.4%). Forty percent of patients (n=28) did not undergo a digital rectal/stomal examination. Only 44% of patients (n=32) had their hernial orifices examined for potentially obstructing hernias. The majority of patients (98.6%) did get an abdominal x-ray, but only 20 patients (27.8%) had serum lactate level measurements.

In terms of patient management (standards 4 and 5, Table 1), 52.8% of patients (n=38) were managed successfully by an entirely conservative approach. In this group, the majority had an appropriate regime (94.7%, n= 36) having intravenous fluid administration, 65.8% (n = 47) and the insertion of a nasogastric tube for decompression with adequate measurements of output). However, only a few patients (5.3%, n = 3)) received Gastrografin as per the Bologna guidelines.

19.4% (n=14) of the patients were managed by laparoscopic adhesiolysis within 24h of admission. Patients who failed conservative treatment, 19.4% (n=11) underwent a laparotomy within 72h, 8.3% (n=5) were surgically managed after 72h of conservative management (mean of 10 days).

Comparing with the standard set, we achieved over 80% inadequate compliance documentation of history and appropriate surgical management. We fared poorly in the documentation of physical signs (31%, n=22) and appropriate use of blood tests and scans (28%, n=20) and usage of Gastrografin (5.3%, n=3) (Table 2).

**Table 2:** Comparsion to standards

Standard	Target (exceptions)	% meeting target
Adequate documentation of patient history	100%	81%
Adequate documentation of physical examination	100%	31%
Investigations: blood tests and scans	100%	28%
Appropriate conservative management	100% (exception – GASTROGRAFIN ALLERGY CONTRA-INDICATIONS TO NG INSERTION)	0% (47% if ignore Gastrografin)
Appropriate surgical management	100%	83%

As an intervention to improve our standards, we created a poster with a flowchart for ASBO management used by the Bologna working group, and we trained our senior and junior doctors about the need to adhere to the flowchart. We liaised with radiologists and radiographers to aid us in the appropriate use of Gastrografin. It was administered orally or via the nasogastric tube at a dose of 100ml with the patient in the erect position. An abdominal x-ray was taken 24h after administering Gastrografin to determine whether it was visible in the colon or not. We also documented whether Gastrografin had acted therapeutically to resolve the obstruction.

Following the introduction of this intervention, we proceeded to re-audit prospectively over 6 months.

### Prospective re-audit

The re-audit identified 60 consecutive patients who were included for analysis. More patients in this group underwent a conservative line of approach, n=48 (80%) and fewer patients n= 4 (6.7%) who were surgically managed as the first line of management when comparing with our preliminary retrospective audit step (52.8% vs. 19.4%, respectively). Even patients who were managed conservatively initially but went on to have surgery within or after 72h were also fewer in number in the re-audit, n = 8 (13.3%) compared to the initial audit. Whether they were managed surgically primarily or after a period of conservative management, the average length of stay was shorter in the re-audit phase (10 days vs. 16 days and 16 days vs. 22 days, respectively). However, this was not the case for patients managed primarily by the conservative approach in the re-audit phase (8 vs. 5 days).

In comparison to the preliminary audit, there appeared to be no change in the way medical history, and physical examination was documented during the re-audit. However, there was a marked difference in the use of appropriate blood tests and CT scans (57% vs. 28%). More patients were being managed conservatively, and fewer patients underwent surgical intervention (Table 3).

**Table 3:** Comparsion to standards

Standard	Target (exceptions)	% meeting target	
		Cycle 1	Cycle 2
Adequate documentation of patient history	100%	81%	78%
Adequate documentation of physical examination	100%	31%	33%
Investigations: blood tests and scans	100%	28%	57%
Appropriate conservative management	100% (exception – GASTROGRAFIN ALLERGY CONTRA-INDICATIONS TO NG INSERTION)	0% (47% if ignore Gastrografin)	10% (40% if ignore Gastrografin)
Appropriate surgical management	100%	83%	67%

We also looked closely at the impact Gastrografin made on ASBO management. As part of the conservative management, more patients received Gastrografin [31/60 (52%) vs. 1/72 (1.4%)] during the re-audit. Nevertheless, only 25 patients (15%) had Gastrografin given as per the protocol with respect to time of administration and dose used. In the remaining 6, Gastrografin was given either late (3/6) or given as an incorrect dose of 30 or 50ml (3/6). However, in 3 out of these 6 patients, the obstruction resolved spontaneously (1 given late, 2 given wrong doses). Five patients out of the 25 (20%) who received Gastrografin as per the protocol, unfortunately, required surgery (1 received 30ml only; 2 were administered at 72h) leaving 20 (80%) who had successful resolution of their obstruction. Overall, there was a significant reduction in the length of hospital stay in those who had received Gastrografin appropriately (5.6 days vs. 10.9 days, p = 0.013).

## Discussion

Postoperative intra-peritoneal adhesions are responsible for the majority of cases of ASBO [10]. The nature of surgery and severity of damage to the peritoneum including the degree of intra-peritoneal contamination are the main risk factors for the development of future adhesions. International guidelines were established in 2010 for the diagnosis and management of ASBO [11]. These guidelines were aimed at reducing the incidence of bowel strangulation by predicting delays in the management of ASBO. Strangulation can be difficult to determine clinically, and any delay in the timing of surgery leaves the patient at a higher risk of bowel resection [8].

The first aim of our audit was to establish if we could improve on appropriate documentation of clinical assessment and management of patients with ASBO. However, we did not notice a change in practice with regards to the documentation of medical history and physical findings when we carried out the re-audit. It is poor practice if a per rectal examination and hernial orifice examination is not part of the clinical assessment in ASBO. This finding in our preliminary audit as well as re-audit is of major concern and should trigger a higher education of staff (particularly junior doctors) within the department. However, it is possible that as per clinical practice, patients were clinically assessed appropriately but if there was no documentation, it was not assumed to have happened. The increased use of serum lactate estimation in the re-audit phase has implied a possible increased awareness in need to identify strangulated bowel. Perhaps the formulation of a dedicated treatment pathway for ASBO may improve documentation and awareness in this situation.

Imaging is a major diagnostic tool for patients who present with suspected ASBO. It is well known that plain abdominal x-rays (supine and erect) showing multiple fluid-filled dilated loops of small bowel suggests small bowel obstruction [12]. All of our patients in both audit and re-audit did have supine plain abdominal x-rays. Even though the use of CT scans are only indicated in making a decision when clinical assessment and plain abdominal x-rays are inconclusive [13], almost all our patients did obtain a CT scan in both phases. This may be because we have easy access to obtaining CT scans quickly, which also helps to rule out other diagnoses. Equally, this may be as a result of inadequate physical examination and over-reliance on CT scans.

Gastrografin is constituted by sodium diatrizoate and meglumine diatrizoate with an osmolality of 2150 mOsm /L. It is postulated that by drawing water into the bowel lumen and reducing mural edema, bowel contractility is stimulated to overcome an obstruction [14,15]. In addition to its apparent therapeutic advantage, Gastrografin has the ability to determine whether an obstruction is settling or not [16,17].

Some authors have observed that Gastrografin reduces the length of hospital stay but not the need for surgical intervention [18–20] whilst others mention a benefit in reducing both hospital stay and need to operate [15; 16, 21–24]. Our re-audit shows a reduction in the need for a laparotomy and an overall reduction in the length of stay. Even though not reaching significance, fewer patients needed eventual surgery during the re-audit in the group that received Gastrografin (14% vs. 34%). It was not possible to analyze this factor in our preliminary audit, as there were hardly any patients who did receive Gastrografin. Even though Gastrografin has been deemed safe, reports of aspiration and anaphylactoid reactions have been documented [25]. Fortunately, we did not have any deaths related to this. One of our patients in the re-audit had a chest infection, but it was difficult to determine whether it had been due to aspiration or hospital-acquired pneumonia.

It has been suggested that prolonged conservative management up to 3 days in the absence of peritonitis/strangulation can be carried out before considering surgery for adhesive small bowel obstruction [5]. The Bologna guidelines incorporate this principle. In comparison to the retrospective audit, fewer patients had surgery after a 72h period of conservative management during the re-audit (8.3% vs. 19.4%). Even in those patients who underwent surgery after a prolonged period of conservative management, i.e. after more than 72h, only 5% had surgery in re-audit compared to 8.3% in the initial audit. Overall more patients were conservatively managed in the re-audit (80% vs. 53%) possibly because of the increased usage of Gastrografin. Fewer patients underwent a laparotomy as primary initial treatment during the re-audit (6.7% vs. 19.4%) implying a changed mindset of the doctor after implementing a flowchart protocol during the re-audit.

## Conclusion

Changes were made successfully during the re-audit and have been implemented in the management of ASBO in this hospital thus closing the audit loop. Significantly, the use of water-soluble contrast medium (Gastrografin) has decreased the need for surgical intervention in this group of patients with small bowel obstruction. Therefore, this strategy should be considered as a valuable, safe and effective approach to the management of ASBO.

## Conflict of Interest

The authors confirm that there are no conflicts of interest.
